# Determinants of exclusive breastfeeding among mothers of infants aged 6 to 12 months in Gwanda District, Zimbabwe

**DOI:** 10.1186/s13006-019-0225-x

**Published:** 2019-07-09

**Authors:** Paddington T. Mundagowa, Elizabeth M. Chadambuka, Pugie T. Chimberengwa, Fadzai Mukora-Mutseyekwa

**Affiliations:** 1 College of Health Agriculture and Natural Sciences, Africa University, Mutare, Zimbabwe; 2 Ministry of Health and Child Care, Gwanda Provincial Hospital, Gwanda, Zimbabwe; 3 Africa University Clinical Research Centre, 132 H. Chitepo Street, Mutare, Zimbabwe

**Keywords:** Exclusive breastfeeding, Practices, Infant, Maternal, Gwanda

## Abstract

**Background:**

In 2016, 98% of children in Zimbabwe received breastmilk, however only 40% of babies under six months were exclusively breastfed 24 h prior to data collection. A 2014 survey revealed that Matabeleland South Province had the country’s highest starvation rates and food insecurities were rife. This study aimed at investigating maternal, infant, household, environmental and cultural factors influencing exclusive breastfeeding (EBF) practice in Gwanda District.

**Methods:**

A cross-sectional study was conducted from January to March 2018. Interviews used pretested structured questionnaires for 225 mothers of infants aged between six and twelve months at immunization outreach points and health facilities. Descriptive statistics, bivariate and multivariate analysis estimated the association between the dependent and independent variables. Exclusive breastfeeding was defined as feeding an infant on breast milk only from birth up to the age of six months.

**Results:**

The majority of mothers (*n* = 193; 89%) had knowledge about EBF and 189 (84%) expressed a positive attitude towards the practice, however, only 81 (36%) practiced exclusive breastfeeding. The most common complementary food/fluid given to the infants was plain water (*n* = 85; 59%). Predictors for EBF were: maternal Human Immuno-deficiency Virus positive status (Odds Ratio [OR] 0.30; 95% Confidence Interval [CI] 0.17, 0.56) and being economically independent (OR 0.41; 95% CI 0.21, 0.79). Barriers to practicing EBF were: being a young mother under 25 years of age (OR 3.05; 95% CI 1.67, 5.57), having one or two children (OR 2.49; 95% CI 1.29, 4.79), living in less than two rooms (OR 3.86; 95% CI 1.88, 7.93) and having a baby of low birthweight (OR 1.05; 95% CI 0.40, 2.71). After multivariate analysis, only the mother’s economic independence was associated with practicing EBF (Adjusted OR [AOR] 0.83; 95% CI 0.30, 0.92). Key informants identified traditional family practices as the major barrier to EBF.

**Conclusion:**

The exclusive breastfeeding rates were low despite the mothers’ high knowledge levels and positive attitudes towards the practice. In addressing the multiple factors influencing the cost effective practice, there is need to channel supportive measures through a system-wide approach. This can be achieved by realigning breastfeeding policy directives as well as community attitudes and values towards the exclusive breastfeeding.

## Background

Exclusive breastfeeding (EBF) refers to the practice of feeding an infant on breast milk alone for the first six months of life without addition of other food or water [1]. World Health Organization (WHO) and United Nations International Children’s Fund (UNICEF) recommend mothers to initiate breastfeeding within the first hour after birth. Thereafter, mothers should continue feeding their babies with breast milk alone (including expressed milk or from a wet nurse) for at least the first six months of life before addition of complementary feeding. Exclusively breastfed infants can only take oral rehydration solution, vitamins and minerals, and prescribed medications. Scientific studies by WHO and UNICEF have proven that breastfeeding is beneficial to both the child and the mother [2]. Breast milk lowers the baby’s risk of sickness from acute and chronic infections and is a cost-effective intervention that reduces infant mortality and morbidity [3, 4].

Many African mothers breastfeed their babies beyond one year but EBF for up to six months is still not commonly practiced [5]. Early introduction of complementary feeding escalates the risk of diarrhea, malnutrition and death [6]. Approximately 22% of infant deaths could be prevented if mothers practiced EBF [7, 8].

In 2016, national statistics showed that 98% of children in Zimbabwe were fed on breastmilk however, only 40% of babies under six months were exclusively breastfed 24 h prior to data collection [9]. According to the Zimbabwe National Nutrition Survey Report of 2018 which used the definition of exclusive breastfeeding as feeding baby on breastmilk only for the past 24 h prior to data collection, Matabeleland South had an EBF rate of 71% [10]. Suboptimal breastfeeding practices in Zimbabwe put most babies at risk of malnutrition [11]. In 2014, Matabeleland South Province had the highest starvation rates in the country and most families survived on one meagre meal per day [12].

In May 2017, the Zimbabwean Ministry of Health and Child Care (MoHCC) carried out a national Lot Quality Assurance Sampling (LQAS) program on nutrition monitoring and Gwanda District was identified as one of the 18 Emergency Districts which were in dire need of infant nutrition monitoring and support [13]. The government and partners such as Global Alliance (GOAL) and UNICEF had versatile Infant and Young Children Feeding (IYCF) programs in the district but provincial median duration for EBF was at 3.7 months instead of six months [14].

This study sought to assess maternal knowledge and attitudes to EBF in Gwanda District. We also sought to determine the maternal and infant related factors as well as household, environmental, psychosocial, cultural factors influencing EBF in the district. Thus, it was pertinent to understand the root causes of the problem by approaching the primary actors in EBF that is, the mothers. We were also motivated to conduct this study in Gwanda District where no similar research has been carried out to date. The urgent need for nutritional support and monitoring in Matabeleland South province prompted the researchers to carry out the study which had the potential to identify factors which encourage or inhibit the uptake of the practice. The significant factors can be used for planning interventions to promote infant nutrition. The study findings could help with the identification of factors which encourage or inhibit the uptake of the practice for planning interventions to promote infant nutrition.

## Methods

### Study design

A community and facility based analytical cross-sectional study was conducted to establish the determinants of EBF practices among mothers who had babies aged six to twelve months in Gwanda District. The research study was conducted over a period of eight weeks from January to March 2018.

### Study setting and participants

The study was carried out in Gwanda District, Matabeleland South Province. The district covers areas around Gwanda town which is 126 km south east of Zimbabwe’s second largest city of Bulawayo along the Bulawayo-Beitbridge highway. The district had 30 health facilities and of these, three were hospitals and 27 were clinics. Gwanda District had an estimated population of 142 943 of which 3,971 were children less than one year and 21,027 children under five years of age. The district had an estimated 32 464 women of child bearing age and 5,775 births were expected annually [15].

### Sample size and sampling technique

The sample size was determined using a study on knowledge and attitudes towards EBF in Iran [16] which assumed the standard deviation of 0.36 within the population of EBF mothers. The sample was calculated using the formula *n* = Z^2^s^2^/e^2^, where ***n*** was the sample size, **Z** was the test statistic for the 95% confidence interval [17]. **S** was the estimated standard deviation of an attribute that was present in the population (assumed to be 0.36) and **e** was the desired precision (0.05). After calculation, the minimum sample size for mothers interviewed was approximately 200. A 10% attrition was anticipated; hence the final adjusted sample size was approximately 223 mothers.

All the 23 health facilities which offer maternal and child health services in the district as well as their respective Extended Program of Immunization Outreach (EPIO) points were selected for the study. Mothers recruited from an EPIO point were regarded as participants for a health facility that covers that catchment area. The district had 54 EPIO points and the researchers targeted EPIO days when mothers converged either at the health facility or EPIO points. Eleven Key Informants comprising of nurses, Village Health Workers (VHWs) and nutritionists were purposively selected for the study.

### Data collection

An interviewer administered questionnaire which was translated to the dominant local language IsiNdebele, was used to collect data from the mothers then back to English for consistency. Key informant interview guides were also designed to cater for both English and IsiNdebele speaking participants (nurses, VHWs and nutritionists). The questionnaire for mothers contained some questions adopted from the Iowa Infant Feeding Attitude Scale (IIFAS) [18].

The research instruments were pretested for validity, reliability and clarity at Gwanda Provincial Hospital. Face to face interviews were carried out in a secluded place or private room to promote confidentiality. Researchers continued to visit the clinic or hospital or EPIO point for interviews on immunization days until the sample size specifically calculated for the health facility was reached. Forty-eight (89%) of the 54 EPIO points as well as two health facilities were visited.

### Study variables

The dependent variable was practicing EBF which refers to feeding the baby on breast milk only from birth up to the age of six months. Mothers who reported correctly practicing EBF were coded ‘EBF’ whilst those who did not were coded ‘non-EBF’. The independent variables for the study were categorized into maternal factors (age, educational level, marital status, health status, knowledge and attitude on EBF, parity, occupation, mode of delivery, HIV status), infant factors (age, health status, sex, birthweight, HIV status), household factors (income source, residence, housing conditions), environmental factors (exposure to media, place of delivery, access to health information), psychosocial and cultural factors (beliefs, religion, self-efficacy).

Mothers with infants 6–12 months of age visiting EPIO points and health facilities during the period of the study were approached for interviews. A written consent form was issued to mothers who met the inclusion criteria and only those who consented to participate in the study were interviewed. Caregivers who were not the mother of the baby and mothers who refused to consent for interviews were also excluded from the study.

### Data analysis

The collected quantitative data were entered and cleaned using Epi Info version 7 before analysis whilst qualitative data was collected from the key informants through use of open-ended questions in the interviewing tool used. The qualitative data obtained were analyzed according to major themes raised during the interviews. Frequencies, means and percentages were used to describe maternal and infant demographic characteristics, knowledge and attitudes on EBF. In measuring maternal knowledge, a correct response was awarded a score of one and an incorrect answer a zero out of a total score of five.

Descriptive statistics, bivariate and multivariate analysis were done in order to ascertain the association between the dependent and independent variables. Multivariate analysis was carried out to measure the strength of interrelationships of several variables at once. Variables such as maternal age, marital status, mode of delivery were inputted simultaneously and compared. A resulting *p* - value of less than 0.05 was considered to be statistically significant.

## Results

### Sociodemographic characteristics

Table 1 shows the maternal sociodemographic characteristics for mothers who participated in the study. The mean age was 26 ± 6 years and 67% of the study participants were in the age group 20–30 years and 64% did not practice EBF. Most mothers were married and had one or two children. The majority were educated to secondary school level, were unemployed and resided in rural areas. Partners/husbands were the major income source in nearly three quarters of the study sample and more than half of the women gave their infants plain water before the age of six months. The majority (99.6%) of the participants in this study fed their babies on breastmilk but the EBF rate was low (36%).

**Table 1:** Maternal sociodemographic characteristics in Gwanda District, Matabeleland South Province, 2018. (*N* = 225)

Variable	Category	^a^Non-EBF		EBF		Total	
		*n* = 144	%	*n* = 81	%	*n* = 225	%
Age (years)	< 20	6	75	2	25	8	4
	20- < 30	96	64	55	36	151	66
	30- < 40	28	49	29	51	57	25
	40- < 50	6	67	3	33	9	5
Median age = 26 ± 6 years							
Marital status	Married	103	65	55	35	158	70
	Cohabiting	17	65	55	35	158	70
	Single	13	54	11	46	24	10
	Separated/divorced	9	53	8	47	17	8
Parity	1–2 children	101	66	52	34	153	68
	3–4 children	37	61	24	39	61	27
	> 4 children	6	55	5	45	11	5
Education level	No formal education	2	100	0	0	2	1
	Primary	22	54	19	46	41	18
	Secondary	118	70	51	30	169	75
	Tertiary	2	15	11	80	13	6
Occupation	Unemployed	117	66	61	34	178	79
	Formal	9	50	9	50	18	8
	Self employed	18	62	11	38	29	13
Religion	Apostolic	67	71	28	29	95	42
	Pentecostal	40	63	24	37	64	29
	Protestant	19	49	20	51	39	17
	None	12	57	9	43	21	9
	Catholic	6	100	0	0	6	3
Residence	Rural	96	62	58	38	154	68
	Urban	26	59	18	41	44	20
	Mine/farm	22	81	5	19	27	12
Number of rooms	1–2	90	76	29	24	119	53
	3–4	42	53	38	47	80	36
	> 4	11	44	14	56	25	11
Income source	Partner/husband	113	69	51	31	164	73
	Self-employed	41	46	24	53	45	20
	Relatives	10	63	6	37	16	7

^a^*Non-EBF* Did not practice Exclusive Breastfeeding

### Maternal knowledge and attitudes on EBF

The majority of women (89%) had knowledge of what exclusive breastfeeding is by definition. Scores on a Likert scale revealed that all interviewed women agreed that breast milk should be the first feed to be given to the baby soon after birth. Ninety-five percent (*n* = 213) agreed that the baby should be put to the breast within an hour of birth and 98% cited that exclusive breastfeeding protects the baby from illnesses. However, 77% (*n* = 111) of those who did not practice EBF and 79% (*n* = 64) of those who practiced EBF their infants disagreed to continuing with breastfeeding when a mother is pregnant.

On an attitude scale (IIFAS), almost all study participants (99%) scored high with regard to EBF being more convenient and beneficial to the baby when compared to formula/mixed feeding. Approximately 90% (*n* = 202) of the women either agreed or strongly agreed that the age of the mother does not influence her ability to practice exclusive breastfeeding. On EBF being the best choice for working mothers, 33% (*n* = 48) of those who did not practice EBF, disagreed when compared to 10% (*n* = 8) of those who practiced EBF and disagreed. Ninety-five percent of the interviewed women either agreed or strongly agreed that breastfed babies are healthier than formula/mixed fed babies. Using the IIFAS, mothers who practiced EBF had a more positive attitude level (*n* = 68; 84%) when compared to mothers who did not.

The Table 2 shows that being a young mother, having one or two children and having a baby of low birthweight was significantly associated with failure to practice whilst mothers with a positive HIV status were more likely to practice EBF.

**Table 2:** Sociodemographic and health related factors influencing EBF in Gwanda District, Matabeleland South Province, 2018 (*N* = 225)

Variable	Non-EBF *n* = 144	EBF *n* = 81	OR (95% CI)	*p* - value
Age: Young mother (less than 25 years old)				
Yes	72	20	1	
No	72	61	3.05 (1.67, 5.57)	0.0002
Marital status: Live with partner				
Yes	120	64	1	
No	22	19	0.62 (0.31, 1.22)	0.17
Parity: Had 1 or 2 children				
Yes	52	15	1	
No	92	66	2.49 (1.29, 4.79)	0.006
Attended formal education				
No	24	19	1	
Yes	120	62	0.65 (0.33, 1.28)	0.21
Maternal HIV status				
Positive	27	35	1	
Negative	117	46	0.30 (0.17, 0.56)	0.0001
Had a chronic disease/condition				
Yes	8	9	1	
No	136	72	0.47 (0.17, 1.30)	0.13
Low birthweight (Below 2500 g)				
Yes	13	7	1	
No	131	74	1.05 (0.40, 2.71)	0.01
Large birthweight (Above 3500 g)				
Yes	31	24	1	
No	113	57	0.65 (0.35, 1.21)	0.17
Sex of the baby				
Boy	81	40	1	
Girl	63	41	0.76 (0.44, 1.31)	0.32
Baby sick for more than 2 weeks when he/she was under 6 months of age				
Yes	27	13	1	
No	117	68	1.21 (0.58, 2.49)	0.61
Infant’s HIV Status				
Positive	2^a^	0^a^	1	
Negative	142	81	0.00	0.00

*OR* Odds Ratio, *CI* Confidence Interval, Chi-square test of association was used to obtain *p* - values and Odds ratios

^a^Cell count is < 5, Chi-square test may not be valid

Table 3 shows that economically independent women were more likely to breastfeed their babies on breast milk only up to the age of six months whilst those who lived in one or two rooms were less likely to practice EBF. Obstetrics and sociocultural factors influencing EBF practice were found not to be significantly associated with the practice as indicated in Table 4. After multivariate analysis, only the mother’s economic independence was associated with practicing EBF (AOR 0.83; 95% CI 0.30, 0.92; *p* < 0.001).

**Table 3:** Household and environmental factors influencing EBF Practices in Gwanda District, Matabeleland South Province, 2018

Variable	Non-EBF *n* = 144	EBF *n* = 81	OR (95% CI)	*p* - value
Maternal occupation				
Employed	27	20	1	
Unemployed	117	61	1.42 (0.73, 2.74)	0.29
Income source				
Independent	21	24	1	
Dependent	123	57	0.41 (0.21, 0.79)	0.007
Residence				
Rural	96	58	1	
Urban/mine	48	23	0.73 (0.44, 1.44)	0.45
Live in few rooms (1 or 2)				
Yes	54	11	1	
No	89	70	3.86 (1.88, 7.93)	0.0001

Chi-square test of association was used to obtain *p* - values and Odds ratios

**Table 4:** Obstetric and sociocultural factors influencing EBF in Gwanda District, Matabeleland South Province, 2018 (N = 225)

Variable	Non-EBF *n* = 144	EBF *n* = 81	OR (95% CI)	*p* - value
Place of birth:				
Home	2^a^	5	1	
Health facility	142	76	0.21 (0.04, 1.12)	0.05
Mode of delivery				
Cesarean	19	15	1	
Vaginal/assisted	124	66	0.67 (0.32, 1.41)	0.29
Baby breastfed in the first hour of birth				
No	64	26	1	
Yes	83	56	1.66 (0.94, 2.93)	0.08
Visited Antenatal Clinic				
No	7	3^a^	1	
Yes	137	78	0.75 (0.19, 3.00)	0.69
Religion				
Apostolic	67	28	1	
Other	77	53	1.65 (0.94, 2.90)	0.08
Treated depressed fontanel using traditional remedies (inkanda)				
Yes	70	29	1	
No	74	52	1.70 (0.97, 2.97)	0.06
Any breastfeeding problems up to 6 months				
Yes	14	5	1	
No	130	76	1.64 (0.57, 4.72)	0.36

^a^Cell count is < 5, Chi-square test may not be valid

Chi-square test of association was used to obtain *p* - values and Odds ratios

## Discussion

The aim of this study was to assess the knowledge and attitudes on exclusive breastfeeding in Gwanda District. We also sought to determine the different maternal, infant, household, environmental, psychosocial and cultural factors influencing EBF practice in this district.

The majority (99.6%) of the participants in this study fed their babies on breastmilk although the EBF rate was low (36%). This EBF rate was lower than the national EBF rate (40%) reported in the Multiple Indicator Cluster Survey (MICS) findings of 2014 and provincial rate reported in the Zimbabwe National Nutrition Survey of 2018 (71%) [10]. The lower estimates of EBF observed in this study were due to the differences in the definitions of EBF since the national surveys used 24 h reporting period rather than the birth to six months period used in this study. The EBF rate we found was higher than studies in Ethiopia (26.4%) and Brazil (15.2%) [19, 20]. Variations in sociodemographic characteristics and cross-cultural preferences may be the cause of differences in the EBF rates.

The interviewed women were knowledgeable on EBF practices, despite the low EBF rate in the Gwanda community. This finding was similar to a study in Nigeria were knowledge was high (82%) but the EBF rate was low (33.5%) [21]. This finding explains why having the knowledge of EBF does not necessarily translates to EBF practice. In most cases, social pressure to introduce complementary feeds tends to outweigh the mother’s knowledge of EBF benefits [22].

Mothers who had one or two children were less likely to exclusively breastfeed their babies when compared to mothers with three or more children. This finding was consistent with study findings in a Jordan study were they concluded that multi-parity was a major predictor of exclusive breastfeeding [23]. Being a novice mother was also found out to be a threat to EBF in two studies conducted in Brazil [20, 24]. The increase in maternal confidence with several prior pregnancies could be due to previous positive experiences in breastfeeding as well as previous negative outcomes observed with early introduction of complementary foods.

Young mothers (less than 25 years of age) were less likely to practice of exclusive breastfeeding in Gwanda District. This finding was similar to observations in a Chinese study were mothers of the age group 15–24 years were less likely to practice EBF due to general traditional practice of prelacteal feeding especially in rural areas [25]. Similarly, a Brazilian study concluded that adolescent mothers were less likely to EBF when compared with older mothers [26]. Being an older mother comes with previous experience, added conviction and commitment to motherhood hence an increased likelihood to EBF the infant [27].

Women who lived in fewer rooms (one or two) were less likely to practice exclusive breastfeeding when compared to those who had and used more than three rooms. Living in fewer rooms can limit the mother’s privacy. Some African cultures view the female breast as a part of a women’s identity and femininity which must remain private, and it is a taboo to expose one’s breasts or openly talk about the breasts [28]. Thus, breastfeeding in the presence of the in-laws or any other respected elders can be viewed as contemptuous behavior. Thus, mothers may not be able to frequently breastfeed in the presence of in-laws or elders due to the limited privacy.

Mothers who were financially independent were more likely to exclusively breastfeed their babies in Gwanda District (OR 0.4; 95% CI 0.21, 0.79; *p* = 0.007). This finding was contrary to studies in which EBF rates were lower among employed mothers when compared to unemployed dependent mothers [19, 29]. Dependence limits the mother’s sense of autonomy. Dependent women often adhere to family opinions on infant feeding because their in-laws or partners dictate what can be given to the baby thus, it is difficult for them to adhere to EBF principles [30].

A maternal HIV positive status was found to be significantly associated with the practice of EBF in Gwanda District. This finding was consistent with studies in South Africa in which they identified that HIV positive women had more knowledge on EBF and were more likely to EBF their babies [31–33]. Mothers who knew their positive HIV status revealed urgency in preventing transmission of the virus to the baby, hence EBF uptake was high among HIV positive mothers [34].

The latest WHO guidelines recommend that health workers encourage EBF to HIV positive mothers [2]. The encouraging uptake or the EBF practice by seropositive mothers shows that the education given to pregnant women by health workers in Gwanda District was consistent with the WHO guidelines. Exclusive breastfeeding by HIV positive women had the potential to reduce mother to child transmission of HIV in Gwanda District.

Babies who had low birthweight (below 2500 g) were 4% less likely to be EBF when compared to babies who had a normal birthweight. This was consistent with a South African study results in which they cited the maternal desire for the infant to gain weight prompting mother’s decision to add complementary feeding before the age of six months [35]. The percentage of children who were given plain water before the age of six months was 58% which is significantly higher than the 28% cited by Zimbabwe Demographic Health Survey (ZDHS 2015) [14]. Our finding was consistent with a study in Binga, Zimbabwe, were mothers gave their young babies plain water with the belief that water would not affect the practice of exclusive breastfeeding [36].

When approaching EBF related issues, health workers need to take into consideration all the perceived threats, barriers and benefits associated with the complex decision to EBF for six months. Such an approach has the potential of improving maternal and infant wellbeing in Gwanda District and other similar settings.

### Study strengths and limitations

By collecting data from mothers with infants who were 6 to 12 months of age, the researchers reduced recall bias. Collecting data from both facilities and the community resulted in more representative data and the use of trained as well as experienced research assistants helped in preserving the quality of the obtained data whilst ensuring proper ethical standards are followed.

The findings of this study cannot be generalized for Matabeleland South Province as well as other settings because participants were only drawn from Gwanda District. The use of convenience sampling method interfered with the representativeness of the collected data and self-reporting may have introduced recall bias to the study. The study was also vulnerable to social desirability bias in which some mothers may have felt EBF to be a more socially acceptable hence, felt compelled to respond positively towards EBF practices. Using the cross-sectional research design made it challenging for the researchers to establish causality. The omission of nutritional status of children (HAZ- stunting, WHZ- wasting and WAZ- underweight) and mothers’ body mass index (BMI) as study variables also limited analysis of the study results.

## Conclusion

The exclusive breastfeeding rates were low despite the mothers’ high knowledge levels and positive attitudes towards the practice. In addressing the multiple factors influencing the cost effective practice, there is need to channel supportive measures through a system wide approach. This can be achieved by realigning breastfeeding policy directives as well as community attitudes and values towards the exclusive breastfeeding. Collaborated efforts from both the healthcare sector and society are vital in promoting and sustaining the optimal breastfeeding practice. This has the potential to improve child health and reducing infant morbidity and mortality in Gwanda District.

## Data Availability

The datasets used and/or analyzed during the current study are available from the corresponding author on reasonable request.

## Abbreviations

CI: Confidence Interval
EBF: Exclusive Breastfeeding
EPIO: Extended Program of Immunization Outreach
GOAL: Global Alliance
HIV: Human Immuno-deficiency Virus
IIFAS: Iowa Infant Feeding Attitude Scale
IYCF: Infant and Young Child Feeding
LQAS: Lot Quality Assurance Sampling
MICS: Multiple Indicator Cluster Survey
MoHCC: Ministry of Health and Child Care
OR: Odds Ratio
UNICEF: United Nations International Children’s Fund
VHWs: Village Health Workers
WHO: World Health Organization
ZDHS: Zimbabwe Demographic Health Survey
